# Prevalence of Hypomagnesemia in ICU Patients at a Tertiary Care Center: A Prospective Observational Study

**DOI:** 10.7759/cureus.81656

**Published:** 2025-04-03

**Authors:** Saurav Shekhar, Raj B Singh, Swapna Lata, Akrity Singh, Ranjeet Rana De, Preeti Sharma, Shivani Sinha

**Affiliations:** 1 Anesthesiology (Trauma and Emergency), Indira Gandhi Institute of Medical Sciences, Patna, IND; 2 Anesthesia and Critical Care, Indira Gandhi Institute of Medical Sciences, Patna, IND; 3 Otolaryngology, Patna Medical College and Hospital, Patna, IND; 4 Community Medicine, Indira Gandhi Institute of Medical Sciences, Patna, IND

**Keywords:** apache ii score, copd, critically ill patients, hypomagnesemia, icu stay, metabolic disturbances, sepsis, ventilator support

## Abstract

Background: Magnesium, the fourth most abundant cation in the human body, plays a crucial role in enzymatic activity, neurochemical transmission, and muscular excitability. Hypomagnesemia in critically ill patients has been associated with neuromuscular dysfunction, cardiovascular complications, metabolic disturbances, and increased morbidity. This study aims to determine the prevalence of hypomagnesemia in critically ill patients and evaluate its impact on disease severity, length of ICU stay, ventilator dependency, and mortality.

Methods: This prospective observational study was conducted in the ICU of a tertiary care hospital after obtaining institutional ethical clearance. A total of 100 critically ill patients aged ≥45 years were enrolled after providing written informed consent. Serum magnesium levels, along with key biochemical markers including serum calcium, serum potassium, procalcitonin, creatinine, blood urea nitrogen (BUN), serum albumin, and Acute Physiology and Chronic Health Evaluation (APACHE) II scores, were documented at admission. Patients with serum magnesium levels >2.6 mg/dL were excluded. The primary outcome was the prevalence of hypomagnesemia (<1.7 mg/dL). Secondary outcomes included ICU stay duration, ventilator days, and 28-day mortality. Statistical analysis was conducted using paired and unpaired t-tests via GraphPad (Dotmatics, Boston, Massachusetts, United States/Insight Venture Management, LLC, New York, New York, United States).

Results: Among 100 patients, 24% had hypomagnesemia (Group A), while 72% had normal magnesium levels (Group B). The mean age in Group A was significantly higher (64.8±6.2 years) compared to Group B (59.2±4.9 years) (p<0.001), indicating an increased prevalence in older patients. Group A showed a slightly higher APACHE II score compared to Group B, signifying increased disease severity. A statistically significant association was found between hypomagnesemia and hypocalcemia (66.7% vs. 26.4%; p<0.001), whereas no clear association was observed between hypomagnesemia and hypokalemia or hypoalbuminemia. Chronic obstructive pulmonary disease (COPD) was significantly more prevalent in Group A than in Group B (p=0.049). Patients with hypomagnesemia had a longer duration of mechanical ventilation (6.8±2.1 vs. 5.9±1.9 days; p<0.05) and prolonged ICU stay (8.36±3.62 vs. 6.8±2.43 days; p<0.05). However, no statistically significant difference in 28-day mortality was observed between the groups (p=0.89).

Conclusion: Hypomagnesemia is highly prevalent among critically ill patients, particularly in older individuals, and is associated with increased disease severity, prolonged ventilator dependency, and extended ICU stay. A strong correlation between hypomagnesemia and hypocalcemia was observed, suggesting the need for comprehensive electrolyte monitoring in critically ill patients. Although hypomagnesemia did not directly influence short-term mortality, its impact on disease progression and ICU morbidity necessitates further investigation. Early detection and timely magnesium supplementation may improve patient outcomes. Larger multi-center studies are warranted to explore the therapeutic implications of magnesium correction in ICU settings.

## Introduction

Magnesium is the fourth abundant crucial cation in the human body and plays a fundamental pivotal role in various tissue functioning at the molecular level. Almost all organ systems of our body need magnesium for proper functioning, and it is the ion re-emphasized in this decade. It is essential for the activity of many enzyme systems and plays an important role regarding neurochemical transmission and muscular excitability [1].

Hypomagnesemia causes severe and potentially life-threatening complications if not diagnosed on time and properly treated and is associated with increased morbidity and mortality with prolonged hospitalization and unfavorable outcomes. Several studies have pointed out that magnesium deficiency may lead to a secondary decrease in potassium and calcium levels in our body and causes critical neuromuscular and cardiovascular clinical manifestations which may cause increased mortality and prolonged hospitalization [2]. Magnesium depletion induces a stress response through the activation of neuroendocrine pathways [3]. The causes of hypomagnesemia in critically ill patients are mainly a result of gastrointestinal disorders or renal loss of magnesium. Magnesium therapy is warranted in few cardiac conditions like torsades de pointes and preeclampsia in pregnant patients. Magnesium administration may be useful to reduce cerebral ischemic events after aneurysmal subarachnoid hemorrhage or in the treatment of severe acute asthma, specifically in children [4]. Deficiencies are accompanied by a variety of structural and functional disturbances. Some of the effects of magnesium on the nervous system are similar to those of calcium. An increased concentration of magnesium in the extracellular fluid causes depression of the central nervous system (CNS). Magnesium has a direct depressant effect on skeletal muscle [5,6].

Regional and demographic factors can significantly affect magnesium levels in critically ill patients. Geographic variations in dietary intake, water mineral content, and socioeconomic status influence baseline magnesium levels. For example, populations with diets low in magnesium-rich foods (e.g., leafy greens, nuts, and whole grains) may be more prone to deficiency [7]. Additionally, critically ill patients from regions with high incidences of gastrointestinal or renal diseases may have a higher prevalence of hypomagnesemia due to increased magnesium losses. Conditions such as diarrhea, chronic kidney disease, and medication use (e.g., diuretics, antibiotics, or chemotherapy) are major contributors to magnesium depletion in ICU settings.

Socioeconomic factors also play a role, as access to healthcare, nutrition, and preventive screenings can affect magnesium levels. Lower-income populations often face dietary deficiencies and delayed medical intervention, increasing their risk for severe hypomagnesemia. Ethnic and genetic variations in magnesium metabolism may further influence susceptibility to deficiency in critical illness [8].

Given its widespread physiological importance, maintaining optimal magnesium levels in critically ill patients is essential. Imbalances, particularly hypomagnesemia, are common in the ICU and have been associated with increased morbidity and mortality. Understanding magnesium's role in critical illness can help guide better management strategies and improve patient outcomes. This study aims to assess the prevalence of hypomagnesemia in critically ill patients as well as the mean number of ventilator days, mean length of ICU stay, and mortality status in such patients.

## Materials and methods

This prospective observational study was done at the Trauma and Emergency ICU of Indira Gandhi Institute of Medical Sciences in Patna, India, after obtaining approval from the institute's Institutional Ethics Committee (approval number: 1157/IEC/IGIMS/2019) and Clinical Trials Registry-India (CTRI) registration (CTRI/2024/12/078372). After written informed consent, 100 critically ill patients admitted to our ICU were included in this study. Serum magnesium, serum calcium, serum potassium, procalcitonin, serum creatinine, blood urea nitrogen (BUN), serum albumin, and Acute Physiology and Chronic Health Evaluation (APACHE) II score were documented at the time of admission. Any co-existing diseases like diabetes, hypertension, kidney disease, liver disease, chronic obstructive pulmonary disease (COPD), thyroid disease, etc. were noted. Primary disease pathology due to which patients were admitted was also recorded.

Inclusion criteria

Included in the study were patients aged ≥45 years, of both sexes, and suffering from kidney failure, cardiac disease, sepsis, COPD, and respiratory complications.

Exclusion criteria

Patients aged <45 years, who refused to participate, and with any neurological illness, traumatic brain injury, a history of trauma leading to current status, a history of alcoholism, and any previous magnesium therapy as well as surgical patients were excluded from our study.

Sample size

Since the study aims to estimate the prevalence of hypomagnesemia in critically ill patients, we use the following formula for sample size estimation in prevalence studies: $n = \frac{Z^2 \times p \times (1 - p)}{d^2}$. Here, n is the required sample size, Z is the Z-score (1.96 for 95% confidence level), p is the estimated prevalence of hypomagnesemia in critically ill patients (from literature [9,10], assumed 30% or 0.30), and d is the margin of error (commonly 0.10 or 10%). Upon calculations, a sample size of 81 patients would be required to estimate prevalence with a 10% margin of error. Since our sample size is 100, it is adequate for prevalence estimation with reasonable precision. Also, using Cohen's effect size approach, 100 patients is reasonable for analyzing major clinical outcomes.

Statistical analysis

Continuous variables are reported as mean±SD, whereas categorical variables are expressed as counts and percentages (%). All statistical analyses were done by paired and unpaired independent t-tests and chi-squared tests using GraphPad (Dotmatics, Boston, Massachusetts, United States/Insight Venture Management, LLC, New York, New York, United States). A p-value of <0.05 is considered significant.

The primary outcome was assessed by the prevalence of hypomagnesemia in critically ill patients, while the secondary outcome was assessed by the mean number of ventilator days, mean length of ICU stay, and mortality status. 

Table 1 depicts the effects of magnesium on various organ systems.

**Table 1:** Effects of magnesium on various organ systems NMDA: N-methyl-D-aspartate

Organ system	Effects
Cardiovascular	Affects the cardiac conduction system
	Influences myocardial excitability and can cause dysarrhythmias
Respiratory	Established role in the treatment of asthma
	Causes bronchodilation and relaxes smooth muscles
	Immunoregulatory effect
Neurovascular	Cerebroprotective effect
	Vasodilator effect maintains healthy blood flow to the brain
	Blocks NMDA receptors
	Decreased level causes neuromuscular hyperexcitability
Endocrine	Few studies show that hypomagnesemia predisposes to increased incidence of type 2 diabetes
	Decreases parathyroid hormones causing hypocalcemia
Bones	Important role in bone homeostasis
	Affects bone formation and influences osteoblastic and osteoclastic activity

## Results

Only four patients presented with increased serum magnesium levels (>2.6 mg/dL) and were therefore excluded from our study. The mean number of patients in Group A (hypomagnesemia) is 24 (24%), whereas in Group B, it is 72 (72%). This signifies a very high prevalence of hypomagnesemia among older, critically ill patients. There is a statistically significant difference in mean age between the two groups (64.8±6.2 years in Group A versus 59.2±4.9 years in Group B), indicating a higher incidence of hypomagnesemia in the older age group (p<0.001). Group A shows a slightly higher APACHE score compared to Group B, suggesting a slightly higher disease severity. COPD is significantly associated with hypomagnesemia (p<0.05), while there is a possible association between hypomagnesemia and hypothyroidism (p=0.05). Magnesium plays a crucial role in smooth muscle relaxation and bronchodilation. Magnesium deficiency can exacerbate bronchoconstriction, making breathing more difficult for COPD patients. Hypomagnesemia contributes to respiratory muscle fatigue, further compromising lung function. Correcting magnesium levels in COPD patients may improve disease outcomes, reduce exacerbations, and enhance overall quality of life [11] (Table 2).

**Table 2:** Demographic profile with primary and secondary outcomes Mg: magnesium; APACHE: Acute Physiology and Chronic Health Evaluation; COPD: chronic obstructive pulmonary disease; CKD: chronic kidney disease; AKI: acute kidney injury

Parameter	Group A (hypomagnesemia)	Group B (normal Mg level)	Statistical test	P-value
Mean number of patients (%)	24 (24%)	72 (72%)	-	-
Mean age (years±SD)	59.8±6.2	53.2±4.9	t-test	<0.001
Gender (male/female)	13/15	43/25	Chi-squared test	0.28
APACHE II score (mean±SD)	23.91±6.8	21.6±5.6	t-test	0.04
Mean serum magnesium level (mg/dL)	1.4±0.21	1.86±0.32	t-test	<0.001
Associated co-existing diseases				
Diabetes	11 (45.8%)	21 (37.2%)	Chi-squared test	0.13
Hypothyroidism	8 (33.3%)	11 (15.3%)	Chi-squared test	0.05
Hypertension	7 (29.2%)	16 (22.2%)	Chi-squared test	0.49
COPD	9 (37.5%)	13 (18.1%)	Chi-squared test	0.04
Cardiac diseases	4 (16.7%)	11 (15.3%)	Chi-squared test	0.80
Kidney failure (CKD/AKI)	6 (25%)	11 (15.3%)	Chi-squared test	0.07
Laboratory findings				
Hypocalcemia (%)	16 (66.7%)	19 (26.4%)	Chi-squared test	<0.001
Hypokalemia (%)	13 (54.2%)	39 (61.9%)	Chi-squared test	0.41
Decreased serum albumin (%)	10 (41.7%)	21 (29.2%)	Chi-squared test	0.21
Secondary outcome				
Mean number of ventilator days	6.8±2.1	5.9±1.9	t-test	0.02
Mean length of ICU stay (days±SD)	8.36±3.62	6.8±2.43	t-test	0.03
Mortality during hospital stay (%)	8 (33.3%)	21 (29.2%)	Chi-squared test	0.89

Based on statistical analysis, there is a significantly strong association between hypomagnesemia and hypocalcemia (66.7% in Group A versus 26.4% in Group B; p<0.001). However, there was no clear association between hypokalemia or hypoalbuminemia and decreased serum magnesium levels. There is a statistically significant difference between Group A and Group B in terms of ventilator days and ICU stay duration. Group A required longer ventilation and had a significantly increased ICU stay duration. However, there was no association between mortality and decreased serum magnesium levels (p=0.89). Other factors such as comorbidities (pulmonary complications, sepsis, cardiovascular disease, renal failure) may play a more dominant role in mortality, overshadowing the effect of magnesium levels. A relatively small sample size might be unable to detect a significant association; therefore, studies with a larger size are required (Table 2).

## Discussion

Magnesium has gained renewed attention in recent times, as magnesium abnormalities are often overlooked in the ICU. Magnesium homeostasis is primarily regulated by the kidneys. In our study, we found a high prevalence of magnesium deficiency among critically ill patients, with a greater occurrence in the older age group. The study group had a higher APACHE score, indicating greater disease severity. Hypomagnesemia was more frequently associated with COPD patients and showed a slight predisposition toward diabetes and hypothyroidism [11-13]. However, we did not find any significant association with other comorbidities. There was a higher incidence of hypocalcemia in the study group. Although patients in the hypomagnesemia group had significantly more ventilator days and longer ICU stays, mortality rates were similar between the two groups.

Limaye and colleagues found that 52% of ICU-admitted patients had hypomagnesemia. They also reported that these patients had an increased mortality rate (57.7% vs. 31.7%), a greater frequency for ventilatory support (73% vs. 53%), a longer duration for mechanical ventilation (4.27 vs. 2.15 days), and a higher prevalence of sepsis, hypocalcemia, and hypoalbuminemia. Additionally, they found that hypomagnesemia was more common in patients with diabetes [14].

Jiang and colleagues, in their systematic review and meta-analysis, also concluded that patients with hypomagnesemia had a higher mortality rate with a greater likelihood of sepsis (RR 2.04; p = 0.0007). There was an increased need for ventilatory support, and the length of ICU stay was also significantly longer in the hypomagnesemia group (RR 1.85; 95% CI 0.43-3.26; p = 0.01) [15].

In another systematic review conducted by Upala and colleagues, critically ill patients with hypomagnesemia had a significantly higher risk of mortality (RR 1.90; 95% CI: 1.48-2.44; p<0.001). The risk of requiring mechanical ventilation was also higher in the hypomagnesemia group (RR 1.65; 95% CI: 1.12-2.43; p=0.01). The length of ICU stay was significantly longer in the hypomagnesemia group, with a mean difference of 4.1 days which was statistically significant [16].

Zeineldin and colleagues found hypomagnesemia in 57% of ICU-admitted patients and an association with eclampsia, seizures, and other conditions (p=0.024). They also observed an increased need for mechanical ventilation and a longer ICU stay [17].

Another study concluded that patients with hypomagnesemia had higher APACHE II scores, longer ICU stays, an increased need for mechanical ventilation, and higher mortality rates. Hypomagnesemia was also associated with a higher incidence of hypokalemia, hypocalcemia, and hypoalbuminemia. Hospital mortality was significantly higher in patients with hypomagnesemia [18].

In one prospective study, researchers found that hypomagnesemia negatively impacts the prognosis of critically ill patients, leading to increased mortality, morbidity, ICU stays, and hospital stays, ultimately contributing to higher healthcare costs and a greater burden on the healthcare system [19].

Soliman et al., in their study, found that patients with hypomagnesemia had higher APACHE II (14.9±5.4 vs. 11.0±6.2) and Sequential Organ Failure Assessment (SOFA) scores (7.1±5.4 vs. 3.9±2.8) at admission, a higher prevalence of severe sepsis and septic shock (57% vs. 11%), an increased ICU stay (15.4±15.5 vs. 2.8±4.7 days), and a higher mortality rate (35% vs. 12%) than other patients [20].

In another study, the incidence of hypomagnesemia was 40.9%, and its association with patient outcomes was found to be statistically significant (p=0.001). Hypomagnesemia was linked to significantly higher mortality (51.3%) compared to normomagnesemia (29.3%) [21]. Hypomagnesemia is 10 times more common in individuals with type 2 diabetes (T2D) than in the general population [22].

A meta-analysis with a large number of participants found a strong association between hypomagnesemia and the risk of all-cause mortality in patients with chronic kidney disease and end-stage renal disease (ESRD) (p<0.00001). They also found a significant association between hypermagnesemia and a reduced risk of cardiovascular mortality (HR 0.71; 95% CI 0.53-0.97; p=0.03) [23].

Limitations of this study

The major limitations of this study are its small sample size and single-center design. However, this pilot study is enough to point out the extent of magnesium deficiency prevalent in critically ill patients. Therefore, a larger multi-centric study with more participants is warranted to assess the often-overlooked causes of prolonged ICU stay. Differences in the prevalence of hypomagnesemia as compared to prior studies might be due to population and geographical variations as well as due to primary disease pathology. Confounding variables like socioeconomic status, underlying chronic conditions, medication use, and other metabolic disorders (like diabetes) may also contribute to hypomagnesemia, complicating the interpretation of results.

## Conclusions

Hypomagnesemia is highly prevalent among critically ill patients, particularly in older individuals, and is associated with increased disease severity, prolonged ventilator dependency, and extended ICU stay. A strong correlation between hypomagnesemia and hypocalcemia was observed, suggesting the need for comprehensive electrolyte monitoring in critically ill patients. Although hypomagnesemia did not directly influence short-term mortality, its impact on disease progression and ICU morbidity necessitates further investigation. Early detection and timely magnesium supplementation may improve patient outcomes. Larger multi-center studies are warranted to explore the therapeutic implications of magnesium correction in ICU settings. We suggest that there should be routine magnesium monitoring in all critically ill patients particularly in the older age group with magnesium supplementation when needed.

## References

[1] Hansen BA, Bruserud Ø (2018). Hypomagnesemia in critically ill patients. J Intensive Care.

[2] Elisaf M, Milionis H, Siamopoulos KC (1997). Hypomagnesemic hypokalemia and hypocalcemia: clinical and laboratory characteristics. Miner Electrolyte Metab.

[3] Cuciureanu MD, Vink R (2011). Magnesium and stress. Magnesium in the Central Nervous System [Internet].

[4] Montupil J, Vincent JL (2012). Magnesium in critical care and anesthesiology [Article in French]. Rev Med Brux.

[5] Tong GM, Rude RK (2005). Magnesium deficiency in critical illness. J Intensive Care Med.

[6] Kothari M, Wanjari A, Shaikh SM (2024). A comprehensive review on understanding magnesium disorders: pathophysiology, clinical manifestations, and management strategies. Cureus.

[7] Mager DR, Wells G (2016). Demographic and lifestyle factors influencing magnesium intake and status. J Nutr Sci Vitaminol.

[8] Tanner RJ, Gropper SS (2014). The influence of geographical location on the magnesium status of populations. J Am Coll Nutr.

[9] Chen M, Sun R, Hu B (2015). The influence of serum magnesium level on the prognosis of critically ill patients [Article in Chinese]. Zhonghua Wei Zhong Bing Ji Jiu Yi Xue.

[10] Ryzen E, Wagers PW, Singer FR, Rude RK (1985). Magnesium deficiency in a medical ICU population. Crit Care Med.

[11] Makwana S, Patel A, Sonagara M (2022). Correlation between serum magnesium level and acute exacerbation in patients with chronic obstructive pulmonary disease (COPD). Cureus.

[12] Gommers LM, Hoenderop JG, Bindels RJ, de Baaij JH (2016). Hypomagnesemia in type 2 diabetes: a vicious circle?. Diabetes.

[13] Wang K, Wei H, Zhang W (2018). Severely low serum magnesium is associated with increased risks of positive anti-thyroglobulin antibody and hypothyroidism: a cross-sectional study. Sci Rep.

[14] Limaye CS, Londhey VA, Nadkart MY, Borges NE (2011). Hypomagnesemia in critically ill medical patients. J Assoc Physicians India.

[15] Jiang P, Lv Q, Lai T, Xu F (2017). Does hypomagnesemia impact on the outcome of patients admitted to the intensive care unit? A systematic review and meta-analysis. Shock.

[16] Upala S, Jaruvongvanich V, Wijarnpreecha K, Sanguankeo A (2016). Hypomagnesemia and mortality in patients admitted to intensive care unit: a systematic review and meta-analysis. QJM.

[17] Zeineldin KH, Kandeel A, Omar E, Sabry S (2022). Effect of hypomagnesaemia on critically ill patients: a retrospective study. Egypt J Crit Care Med.

[18] Madyalkar S, Mahesh E, Gurudev KC (2025). WCN25-2384: a study of hypomagnesemia on clinical outcome of critically ill patients in medical intensive care unit in a tertiary care centre. Kidney Int Rep.

[19] Santosh Raju K, BhaskaraRao JV, Naidu BT, Sunil Kumar N (2023). A study of hypomagnesemia in patients admitted to the ICU. Cureus.

[20] Soliman HM, Mercan D, Lobo SS, Mélot C, Vincent JL (2003). Development of ionized hypomagnesemia is associated with higher mortality rates. Crit Care Med.

[21] Gonuguntla V, Talwar V, Krishna B, Srinivasan G (2023). Correlation of serum magnesium levels with clinical outcome: a prospective observational study in critically ill patients admitted to a tertiary care ICU in India. Indian J Crit Care Med.

[22] Oost LJ, Tack CJ, de Baaij JH (2023). Hypomagnesemia and cardiovascular risk in type 2 diabetes. Endocr Rev.

[23] Xiong J, He T, Wang M (2019). Serum magnesium, mortality, and cardiovascular disease in chronic kidney disease and end-stage renal disease patients: a systematic review and meta-analysis. J Nephrol.

